# Epidemiological situation, laboratory capacity and preparedness for carbapenem-resistant *Acinetobacter baumannii* in Europe, 2019

**DOI:** 10.2807/1560-7917.ES.2020.25.45.2001735

**Published:** 2020-11-12

**Authors:** Felix Lötsch, Barbara Albiger, Dominique L. Monnet, Marc J. Struelens, Harald Seifert, Anke Kohlenberg, European Antimicrobial Resistance Genes Surveillance Network (EURGen-Net) carbapenem-resistant Acinetobacter baumannii capacity survey group, EURGen-Net carbapenem-resistant Acinetobacter baumannii capacity survey group, EURGen-Net carbapenem-resistant Acinetobacter baumannii capacity survey group, Petra Apfalter, Rainer Hartl, Te-Din Daniel Huang, Olivier Denis, Stefana Sabtcheva, Ivan N. Ivanov, Arjana Tambić Andrašević, Irina Pristaš, Panagiota Maikanti, Despo Pieridou, Jaroslav Hrabak, Helena Žemličková, Anette M. Hammerum, Louise Roer, Marika Jürna-Ellam, Jari Jalava, Kati Räisänen, Anaïs Potron, Patrick Plésiat, Niels Pfennigwerth, Spyros Pournaras, Alkiviadis Vatopoulos, Ákos Tóth, Andrea Kurcz, Kristján Orri Helgason, Karen Burns, Monica Monaco, Giulia Errico, Oksana Savicka, Aistė Mierauskaitė, Monique Perrin, Alexandre Mzabi, Elizabeth Anne Scicluna, Nina Nestorova, Leo M. Schouls, Ørjan Samuelsen, Oliver Kacelnik, Dorota Żabicka, Manuela Caniça, Vera Manageiro, Irina Codita, Gabriel Popescu, Eva Schréterová, Andrea Žáková, Mateja Pirš, Jesús Oteo-Iglesias, Belén Aracil, Karin Sjöström, Petra Edquist, Katie L. Hopkins, Jane Turton, Christopher Nugent, Alistair Leanord, Mandy Wootton, Mari Morgan, Andi Koraqi, Artan Bego, Maja Travar, Arsim Kurti, Lul Raka, Vineta Vuksanović, Milena Lopičić, Ana Kaftandjieva, Ivana Ćirković, Deana Medic, Serap Süzük Yildiz, Hüsniye Şimşek

**Affiliations:** 1 European Centre for Disease Prevention and Control (ECDC), Stockholm, Sweden; 2 Institute for Medical Microbiology, Immunology and Hygiene, University of Cologne, Cologne, Germany; 3 German Center for Infection Research (DZIF), partner site Bonn-Cologne, Germany; 4 The EURGen-Net carbapenem-resistant *Acinetobacter baumannii* capacity survey group members are listed below

**Keywords:** Acinetobacter baumannii, carbapenem resistance, Whole Genome Sequencing, laboratory capacity, Surveillance, Europe

## Abstract

To update information on the epidemiological situation and national capacity for detection, surveillance and containment of carbapenem-resistant *Acinetobacter baumannii* (CRAb) in Europe, we performed a survey in 37 countries. Nine countries reported regional or inter-regional spread and seven an endemic situation. Laboratories with a reference function, surveillance systems, and a national containment plan for CRAb existed in 30, 23 and eight countries, respectively. A pan-European molecular survey would provide in-depth understanding of the CRAb epidemiology.

To better understand the current epidemiological situation of carbapenem-resistant *Acinetobacter baumannii* (CRAb) in Europe and the surveillance and control activities in invididual countries, we conducted a survey in 37 countries. The main aims were (i) to update the information about the epidemiological stages of spread of CRAb in Europe, and (ii) to assess the current national capacity for laboratory detection, identification and characterisation, surveillance and containment of CRAb.

## Terminology

Most nosocomial outbreaks are caused by *A. baumannii* sensu stricto (s.s.), which is characterised by its ability to survive long periods of time on dry surfaces [1], while outbreaks of other closely related species are rare. In this report and unless specified otherwise, ‘*A. baumannii*’, or ‘Ab’ as in CRAb, refer to *A. baumannii* s.s. as a species (and not the *A. calcoaceticus-A. baumannii* complex or the *A. baumannii* group).

## Epidemiological situation

A questionnaire using the EUSurvey online tool was sent to the EURGen-Net national coordinators from 37 countries in December 2019. The questions in this survey about the situation in 2019 were adapted from two previous assessments of CRAb in Europe, one in 2013 for that same year [2] and one in 2015 covering 2014 and the beginning of 2015. To assess the epidemiological stage of CRAb dissemination across each country, a previously used seven-stage scale (0, 1, 2a, 2b, 3, 4 and 5) was applied [3]. All contacted coordinators completed the survey, with individual answers received from the United Kingdom (UK) for England, Northern Ireland, Scotland and Wales. While the data for these four countries in the UK are shown separately in some of the tables of the current report, all analyses count the United Kingdom as a single country. 

With regards to the epidemiological stage of spread in 2019, 10 countries reported sporadic, unrelated cases (*stage 1*), two reported single hospital outbreaks (*stage 2a*), eight reported sporadic hospital outbreaks (*stage 2b*), five reported regional spread (*stage 3*), four reported inter-regional spread (*stage 4*), and seven reported an endemic situation (*stage 5*). For one country the stage was uncertain. The epidemiological stages by country are shown in the Figure and a comparison with the results from previous similar surveys is provided in  Table 1. Compared to the result of the 2015 survey, there was a decrease of the epidemiological stage in 13 countries, an identical stage in 16 countries, and an increase in five countries. No comparison could be made for three countries as no epidemiological stage was available from the 2015 study ( Table 1).

**Figure:**
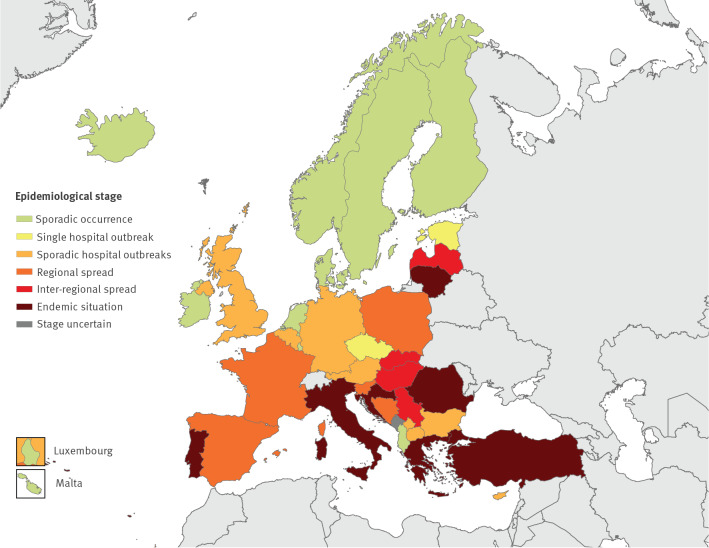
Epidemiological situation of carbapenem-resistant *Acinetobacter baumannii*, assessment by national experts in European countries, 2019 (n = 37)

Administrative boundaries: EuroGeographics, UN-FAO.

**Table 1:** Comparison of epidemiological stages of carbapenem-resistant *Acinetobacter baumannii* in European countries, 2013–2019 (n = 37)

Country	Epidemiological stage of spread of CRAb			Change between 2014–15 and 2019
	2013 [**2**]	2014–15 (previously unpublished)	2019	
Albania	Stage 1	Stage 1	Stage 1	→
Austria	Stage 1	Stage 1	Stage 2b	↑
Belgium	Stage 3	Stage 2b	Stage 2b	→
Bosnia and Herzegovina^a^	Stage 1	Stage 3	Stage 3	→
Bulgaria	Stage 2b	Stage 2a	Stage 2b	→
Croatia	Stage 5	Stage 5	Stage 5	→
Cyprus	Stage 3	Stage 4	Stage 2a	↓
Czechia	Stage 4	Stage 2b	Stage 2b	→
Denmark	Stage 2b	Stage 1	Stage 1	→
Estonia	Stage 2a	Uncertain	Stage 2a	NA
Finland	Stage 1	Stage 2a	Stage 1	↓
France	Stage 3	Stage 2b	Stage 3	↑
Germany	Stage 4	Stage 3	Stage 2b	↓
Greece	Stage 5	Stage 5	Stage 5	→
Hungary	Stage 4	Stage 4	Stage 4	→
Iceland	Stage 0	Stage 1	Stage 1	→
Ireland	Stage 2a	Stage 1	Stage 1	→
Italy	Stage 5	Stage 5	Stage 5	→
Kosovo^b^	Stage 3	Stage 5	Stage 2b	↓
Latvia	Stage 5	Stage 2b	Stage 4	↑
Lithuania	Stage 5	Uncertain	Stage 5	NA
Luxembourg	Stage 1	Stage 0	Stage 1	↑
Malta	Stage 1	Stage 2a	Stage 1	↓
Montenegro	Stage 0	Uncertain	Uncertain^c^	NA
Netherlands	Stage 1	Stage 2a	Stage 1	↓
North Macedonia	Stage 1	Stage 1	Stage 2b	↑
Norway	Stage 1	Stage 2a	Stage 1	↓
Poland	Stage 2b	Stage 4	Stage 3	↓
Portugal	Stage 4	Stage 5	Stage 5	→
Romania	Stage 2b	Stage 5	Stage 5	→
Serbia	Stage 2b	Stage 5	Stage 4	↓
Slovakia	Stage 4	Stage 4	Stage 4	→
Slovenia	Stage 2a	Stage 4	Stage 3	↓
Spain	Stage 3	Stage 4	Stage 3	↓
Sweden	Stage 2a	Stage 2a	Stage 1	↓
Turkey	Stage 2b	Stage 5	Stage 5	→
United Kingdom^d^	Stage 4	Stage 4	Stage 2b	↓

CRAb: carbapenem-resistant *Acinetobacter baumannii*. NA: not applicable.

The epidemiological stages of carbapenem-resistant *Acinetobacter baumannii* are defined as follow: stage 0: no cases reported; stage 1: sporadic occurrence (epidemiologically-unrelated single cases); stage 2a: single hospital outbreak (two or more epidemiologically-associated cases with indistinguishable geno- or phenotype in a single institution); stage 2b: sporadic hospital outbreaks (unrelated hospital outbreaks with epidemiologically unrelated introduction or different strains, no autochthonous inter-institutional transmission reported); stage 3: regional spread (more than one epidemiologically-related hospital outbreak confined to hospitals that are part of the same region or health district, indicating regional autochthonous inter-institutional transmission); stage 4: inter-regional spread (multiple epidemiologically-related outbreaks occurring in different health districts, indicating inter-regional autochthonous inter-institutional transmission); stage 5: endemic situation (most hospitals in a country are repeatedly seeing cases admitted from autochthonous sources).

^a^ The results reported for Bosnia and Herzegovina only apply to the Republic of Srpska.

^b^ This designation is without prejudice to positions on status, and is in line with United Nations Security Council resolution 1244/99 and the International Court of Justice Opinion on the Kosovo declaration of independence.

^c^ *Acinetobacter* spp. and *Pseudomonas* spp. are becoming a major problem in Montenegro as they are frequently isolated and are increasingly resistant to carbapenems. However, a National Reference Laboratory for these bacteria does not exist and all answers in the manuscript are connected with that fact. Therefore, it was judged that a realistic picture of the situation cannot be presented.

^d^ The reported results are for the United Kingdom overall. The epidemiological stages vary among countries within the United Kingdom.

## National surveillance and notification of cases as well as control guidelines

A national system for surveillance of CRAb, either mandatory or voluntary, was in place in only 23 of the 37 countries (Table 2) with establishment of surveillance systems in only four additional countries since the previous survey, indicating that surveillance has not been further expanded and that attention and resources might have shifted away from CRAb, possibly with a focus on the spread of carbapenem-resistant Enterobacterales in many European countries [3]. Notification of cases was mandatory or recommended in 14 countries compared to nine in the period 2014 to 2015. 

**Table 2:** National capacity for surveillance and containment of carbapenem-resistant *Acinetobacter baumannii*, European countries, 2019 (n = 37)

Country		NRL	Routine referral of suspected CRAb isolates to NRL	National system for surveillance	National recommendation or obligation for notification	National guideline on phenotypic carbapenem susceptibility testing	National guideline on molecular characterisation of carbapenem resistance	National recommendation or guideline on infection control measures for CRAb	National plan for containment of CRAb	National policy or guideline on screening
**Albania**		Yes	No	No	No	No	No	No	No	No
**Austria**		Yes	Subset of isolates	Voluntary	No	No	No	No	No	Selective screening
**Belgium**		Yes	Subset of isolates	Mandatory	Yes	Yes	No	For single cases and outbreaks	Yes	Selective screening
**Bosnia and Herzegovina**^a^		No	NA	No	No	No	No	No	No	No
**Bulgaria**		Yes	Subset of isolates	Voluntary	No	Yes	No	For single cases and outbreaks	In preparation	No
**Croatia**		No	NA	Mandatory	No	No	No	In preparation for single cases	No	Selective screening
**Cyprus**		No	NA	Voluntary	No	No	No	No	No	No
**Czechia**		Expert laboratory^b^	Subset of isolates	No	No	No	No	No	No	No
**Denmark**		Yes	All isolates	Mandatory	No	Yes	No	For single cases and outbreaks	Yes	Selective screening
**Estonia**		No	NA	No	In preparation	No	No	No	No	No
**Finland**		Expert laboratory^b^	Subset of isolates	Mandatory	Yes	No	Yes	For single cases and outbreaks	Yes	Selective screening
**France**		Yes	All isolates	Voluntary	No	Yes	No	No	No	No
**Germany**		Yes	Subset of isolates	Mandatory	Yes	No	No	For single cases	No	No
**Greece**		No	NA	Voluntary	Yes, for bacteraemias	No	No	For single cases and outbreaks	Yes	No
**Hungary**		Yes	Subset of isolates	Mandatory	Yes	Yes	No	For single cases and outbreaks	In preparation	Selective screening
**Iceland**		Expert laboratory^b^	All isolates	Mandatory	Yes	Yes	Yes	For single cases and outbreaks	Yes	Selective screening
**Ireland**		Yes	Subset of isolates	Mandatory	Yes	Yes	Yes	For single cases	No	No
**Italy**		Expert laboratory^b^	No	Voluntary	No	No	No	Yes^c^	No	No
**Kosovo^d^**		Expert laboratory^b^	All isolates	No	No	No	No	No	No	No
**Latvia**		Expert laboratory^b^	No	Yes	Yes	No	No	No answer	No answer	No
**Lithuania**		Expert laboratory^b^	Subset of isolates	Mandatory	No	No	No	No	No	No
**Luxembourg**		Expert laboratory^b^	All isolates	No	No	No	No	No	No	No
**Malta**		Expert laboratory^b^	All isolates	Voluntary	Other^e^	No	No	Yes^e^	No	No
**Montenegro**		No	NA	No	No	No	No	No	No	No
**The Netherlands**		Expert laboratory^b^	No	No	No	No	No	Yes	No	No
**North Macedonia**		Expert laboratory^b^	No	Yes^f^	No	No	No	No^g^	No	No
**Norway**		Yes	All isolates	Mandatory	Yes	Yes	No	For single cases and outbreaks	Yes	Selective screening
**Poland**		Expert laboratory^b^	Subset of isolates	No	Yes	Yes	No	No	No	No
**Portugal**		Yes	Subset of isolates	Mandatory	Yes	Yes	No	In preparation	In preparation	No
**Romania**		Expert laboratory^b^	Subset of isolates	Voluntary	No	No	No	In preparation	No	No
**Serbia**		Yes	Subset of isolates	In preparation	No	No	No	No	No	No
**Slovakia**		No	NA	No	Yes	No	No	No	No	No
**Slovenia**		Expert laboratory^b^	No	No	No	Yes	No	No^h^	No	No
**Spain**		Expert laboratory^b^	Subset of isolates	In preparation	In preparation	Yes	No	In preparation	Yes	No
**Sweden**		Yes	All isolates	Voluntary	Yes	Yes	No	Yes	Yes	Selective screening
**Turkey**		Yes	No	Voluntary	Yes	Yes	No	For outbreaks	No	Selective screening
**UK**	**England**	Yes	Subset of isolates	Voluntary	In preparation	Yes	No	For outbreaks	No	Yes
	**Northern Ireland**	Yes	Subset of isolates	No	No	Yes	Do not know	Other^h^	No	No
	**Scotland**	Yes	All isolates	Voluntary	Yes	Yes	No	For single cases and outbreaks	Yes	No
	**Wales**	Yes	All isolates	In preparation	No	Yes	No	Other^i^	No	Selective screening

CRAb: carbapenem-resistant *Acinetobacter baumannii*; NA: not applicable; NRL: national reference laboratory; UK: United Kingdom.

^a^ Results reported for Bosnia and Herzegovina only apply to Republic of Srpska.

^b^ An expert laboratory fulfils the role of a NRL.

^c^ Regional or local recommendations/guidelines apply.

^d^ This designation is without prejudice to positions on status, and is in line with United Nations Security Council resolution 1244/99 and the International Court of Justice Opinion on the Kosovo declaration of independence.

^e^ All organisms are isolated in one laboratory, which is interfaced with the surveillance unit.

^f^ There is a national system for surveillance of multidrug-resistant microorganisms, but not specifically for CRAb. It is an obligation to report these microorganisms through the Centers of Public Health to the National Institute of Public health.

^g^ There is no national recommendation or guideline on infection control measures for CRAb, but World Health Organization guidelines are followed.

^h^ Guidelines available at institutional level.

^i^ Part of the general policy on multidrug-resistant organisms.

Progress was made in developing national recommendations or guidelines on infection control measures for CRAb, with 15 countries having guidelines in 2019 compared to 12 countries in the 2014 to 2015 period. A national plan for containment of CRAb existed in eight countries; however, six of the seven CRAb endemic countries did not have such a plan. National guidelines on phenotypic carbapenem susceptibility testing were present in 15 countries, whereas a national guideline on molecular characterisation of carbapenem resistance was available in only three countries. Ten countries had a national screening policy or guideline. For admission screening of patients to acute care hospitals, all of these guidelines recommended selective screening in high-risk situations (outbreaks) or of high-risk groups, for example after hospitalisation abroad. The survey did not include any questions regarding the laboratory capacity and microbiological methods used for screening.

## National laboratory capacity

Collection of data on isolates analysed by standardised microbiological methods is crucial for a reliable assessment of the spread of CRAb. However, laboratories with a reference function for CRAb existed in only 30 of the 37 countries. Differentiation between *A. baumannii* s.s. and other closely related species, such as *A. pittii*, *A. nosocomialis*, *A. dijkshoorniae* or *A. seifertii*, has been considered difficult in the past, but has improved with the widespread availability of matrix-assisted laser desorption/ionization-time of flight mass spectrometry (MALDI-TOF/MS) method for species discrimination [4]. Widespread use of this technology in clinical laboratories is reflected in the answers indicating that, in 11 countries, all or nearly all clinical laboratories routinely use MALDI-TOF/MS for species identification; however, on the other end of the scale, experts from 13 countries reported that no or very few clinical laboratories in their country have access to MALDI-TOF/MS. In the national reference or expert laboratories, the most common method for species identification was MALDI-TOF/MS (n = 22) followed by PCR / gene-sequencing (n = 15).

The most frequently used methods for determination of phenotypic carbapenem susceptibility were disk diffusion (n = 23), gradient tests (n = 19) and commercial broth microdilution (n = 17). European Committee on Antimicrobial Susceptibility Testing (EUCAST) breakpoints for susceptibility testing were used in all countries with a laboratory with reference function. Most national reference or expert laboratories (25 of 30 laboratories) reported that they perform genotypic characterisation of carbapenem resistance. The most commonly used methods were PCR (n = 18) and whole genome sequencing (n = 16).

Colistin susceptibility testing of *A. baumannii* isolates was performed in 25 of the 30 reference or expert laboratories with broth microdilution according to EUCAST recommendations (four laboratories did not use broth microdilution and the information was missing for one laboratory). Of the 25 laboratories using broth microdilution for colistin testing, nine tested all referred *A. baumannii* isolates for colistin susceptibility and the remaining 16 only tested a subset of isolates. The survey did not include questions regarding internal and external quality assessment in the laboratories. Detailed information on the laboratory capacity is provided in Table 3.

**Table 3:** Methods used by national reference or expert laboratories for species identification, phenotypic carbapenem susceptibility testing and genotypic characterisation of carbapenem-resistant *Acinetobacter baumannii* (CRAb), European countries, 2019 (n = 37)

Country		Method for species determination				Method for phenotypic carbapenem susceptibility testing						Method for genotypic characterisation of carbapenem resistance				
		Biochemical	MALDI-TOF	PCR/gene sequencing	WGS	Automated system	Disc diffusion	Gradient test	Commercial broth microdilution	Agar dilution	In-house broth microdilution	PCR	Real-time PCR	Single-gene sequencing	WGS	Other
Albania		Yes	No	No	No	Yes	Yes	Yes	No	No	No	Not performed				
Austria		Yes	Yes	Yes	No	No	Yes	Yes	Yes	No	No	Yes	No	No	No	Yes^a^
Belgium		No	Yes	Yes	Yes	No	Yes	No	Yes	No	No	Yes	No	Yes	Yes	No
Bosnia and Herzegovina^b^		No NRL or expert laboratory														
Bulgaria		Yes	No	Yes	No	No	Yes	No	Yes	No	No	Yes	No	No	No	No
Croatia		No NRL or expert laboratory														
Cyprus		No NRL or expert laboratory														
Czechia		Yes	Yes	No	No	No	Yes	Yes	Yes	No	No	Yes	No	Yes	Yes	No
Denmark		No	Yes	Yes	Yes	No	Yes	Yes	Yes	Yes	No	No	No	No	Yes	No
Estonia		No NRL or expert laboratory														
Finland		No	No	No	Yes	Yes	Yes	Yes	No	No	No	No	No	No	Yes	No
France		No	Yes	Yes	No	No	Yes	Yes	Yes	No	Yes	Yes	No	Yes	Yes	No
Germany		No	Yes	Yes	No	No	Yes	Yes	Yes	No	No	Yes	No	Yes	Yes	No
Greece		No NRL or expert laboratory														
Hungary		No	Yes	Yes	No	No	Yes	Yes	No	No	Yes	Yes	No	No	Yes	No
Iceland		No	Yes	Yes	No	No	Yes	Yes	Yes	No	No	Yes	Yes	No	Yes	Yes^c^
Ireland		No	Yes	No	No	No	No	Yes	No	No	No	No	Yes	No	No	No
Italy^d^		Yes	No	Yes	Yes	No	Yes	Yes	Yes	No	No	Yes	No	Yes	Yes	No
Kosovo^e^		Yes	No	No	No	Yes	Yes	Yes	Yes	No	No	Not performed				
Latvia		Yes	Yes	No	No	Yes	No	Yes	No	No	No	No	Yes	No	No	No
Lithuania		No	Yes	No	No	No	Yes	No	Yes	No	No	Not performed				
Luxembourg		No	Yes	No	No	No	Yes	No	Yes	No	No	No	Yes	No	Yes	No
Malta		No	Yes	No	No	Yes	No	No	No	No	No	Not performed				
Montenegro		No NRL or expert laboratory														
The Netherlands		No	Yes	No	No	Yes	No	No	No	No	No	Yes	No	No	Yes	No
North Macedonia		Yes	N	No	No	Yes	Yes	Yes	No	No	No	No	Yes	No	No	No
Norway		No	Yes	Yes	Yes	No	No	No	Yes	No	No	Yes	No	No	Yes	No
Poland		Yes	No	No	No	No	Yes	Yes	No	No	Yes	Yes	No	No	No	No
Portugal		No	Yes	Yes	No	No	Yes	No	No	No	Yes	Yes	No	Yes	Yes	No
Romania		Yes	Yes	Yes	No	No	Yes	No	No	No	No	Yes	No	Yes	No	No
Serbia		No	Yes	No	No	No	Yes	Yes	Yes	No	No	Yes	No	No	No	No
Slovakia		No NRL or expert laboratory														
Slovenia		No	Yes	Yes	No	No	Yes	Yes	Yes	No	No	Yes	Yes	No	Yes	No
Spain		Yes	Yes	Yes	Yes	No	Yes	Yes	Yes	No	Yes	Yes	Yes	Yes	Yes	No
Sweden		No	Yes	No	Yes	No	No	No	Yes	No	No	No	No	No	Yes	No
Turkey		No	Yes	Yes	No	No	Yes	Yes	No	Yes	Yes	Yes	No	No	No	No
UK	England	No	Yes	Yes	No	No	No	Yes	Yes	No	No	Yes	Yes	No	No	No
	Northern Ireland	See UK – England														
	Scotland	Yes	No	No	No	No	No	No	Yes	No	No	Not performed				
	Wales	No	Yes	No	Yes	No	Yes	Yes	No	No	Yes	Yes	Yes	No	Yes	No

MALDI-TOF: matrix-assisted laser desorption/ionization-time of flight mass spectrometry; NRL: national reference laboratory; PCR: polymerase chain reaction; UK: United Kingdom; WGS: whole genome sequencing.

^a^ Commercial multiplex nucleic acid amplification methods.

^b^ Results reported for Bosnia and Herzegovina only apply to Republic of Srpska.

^c^ Commercial loop-mediated isothermal amplification.

^d^ Species determination, phenotypic and genotypic characterisation are not routinely performed but only upon request.

^e^ This designation is without prejudice to positions on status, and is in line with United Nations Security Council resolution 1244/99 and the International Court of Justice Opinion on the Kosovo declaration of independence.

## Ethical statement

This study did not involve personal data and ethical approval was thus not required. The release of the included national data was approved by the authors.

## Discussion

CRAb poses a significant threat to patients and healthcare systems in countries of the European Union (EU)/European Economic Area (EEA) [5] with an estimated 2,363 annual attributable deaths in 2015 [6]. According to 2018 data from the European Antimicrobial Resistance Surveillance Network (EARS-Net), nearly a third of invasive *Acinetobacter* spp. isolates in the EU/EEA are already resistant to carbapenems, limiting the availability of adequate treatment [7]. Several reports on outbreaks of CRAb in European countries have recently been published, possibly indicating increasing spread [8-12]. These outbreaks frequently affect intensive care units, and morbidity and mortality are high. In the current study however, our results show that perception of the epidemiological situation of CRAb has not changed substantially since the last survey conducted in 2015. The extent of spread of CRAb appeared to be decreasing in some countries while it was increasing in other countries. The underlying reasons for these developments are not clear. On the one hand, decreasing epidemiological stages may reflect successful local or national control efforts. Control programmes were shown to be effective both in settings of high endemicity [13] and outbreaks [14,15]. On the other hand, the decrease in the epidemiological stage was not always confirmed by the carbapenem resistance rates in invasive isolates reported by EARS-Net or the Central Asian and European Surveillance of Antimicrobial Resistance network (CAESAR) [7,16]. Some countries reported a low epidemiological stage of spread of CRAb whereas EARS-Net showed that, in 2018, a high or very high proportion of *Acinetobacter* spp. invasive isolates in the same country were resistant to carbapenems [7]. One possible explanation for such discrepancies could be that local outbreaks of CRAb are driving the national resistance proportions in *Acinetobacter* spp. invasive isolates as reported to EARS-Net. In addition, two countries also mentioned increasing numbers of carbapenem-resistant isolates in *Acinetobacter* species other than *A. baumannii* s.s., e.g. *A. pittii* or *A. lwoffii* that were not included in this assessment of epidemiological stages but are included in the EARS-Net collection of data on *Acinetobacter* species. Finally, the epidemiological stages were determined by a self-assessment of the national representatives and might have been affected by unavailability of national surveillance data or reference laboratories in some countries, especially as differentiation between stages might be difficult without molecular typing results. The three surveys over time were also not always answered by the same national expert and it cannot be excluded that changes resulted from differences in subjective judgement.

An interest to participate in a European structured survey including the collection of CRAb isolates or whole genome sequencing data thereof as well as related epidemiological information was expressed by 32 countries. Such a survey would help identify successful clones and predominant lineages and the extent of their spread, provide a better understanding of predominant resistance mechanisms to carbapenems and other antimicrobials and allow conclusions on potential cross-border spread of CRAb.

## EURGen-Net carbapenem-resistant *Acinetobacter baumannii* capacity survey group

Austria: Petra Apfalter and Rainer Hartl; Belgium: Te-Din Daniel Huang and Olivier Denis; Bulgaria: Stefana Sabtcheva and Ivan N. Ivanov; Croatia: Arjana Tambić Andrašević and Irina Pristaš; Cyprus: Panagiota Maikanti and Despo Pieridou; Czechia: Jaroslav Hrabak and Helena Žemličková; Denmark: Anette M. Hammerum and Louise Roer; Estonia: Marika Jürna-Ellam; Finland: Jari Jalava and Kati Räisänen; France: Anaïs Potron and Patrick Plésiat; Germany: Niels Pfennigwerth; Greece: Spyros Pournaras and Alkiviadis Vatopoulos, Hungary: Ákos Tóth and Andrea Kurcz; Iceland: Kristján Orri Helgason; Ireland: Karen Burns; Italy: Monica Monaco and Giulia Errico; Latvia: Oksana Savicka; Lithuania: Aistė Mierauskaitė; Luxembourg: Monique Perrin and Alexandre Mzabi; Malta: Elizabeth Anne Scicluna and Nina Nestorova; Netherlands: Leo M. Schouls; Norway: Ørjan Samuelsen and Oliver Kacelnik; Poland: Dorota Żabicka; Portugal: Manuela Caniça and Vera Manageiro; Romania: Irina Codita and Gabriel Popescu; Slovakia: Eva Schréterová and Andrea Žáková; Slovenia: Mateja Pirš; Spain: Jesús Oteo-Iglesias and Belén Aracil; Sweden: Karin Sjöström and Petra Edquist; United Kingdom (UK)-England: Katie L. Hopkins and Jane Turton, UK- Northern Ireland: Christopher Nugent; UK – Scotland: Alistair Leanord; UK-Wales: Mandy Wootton and Mari Morgan; Albania: Andi Koraqi and Artan Bego; Bosnia and Herzegovina: Maja Travar; Kosovo^a^: Arsim Kurti and Lul Raka; Montenegro: Vineta Vuksanović and Milena Lopičić ; North Macedonia: Ana Kaftandjieva; Serbia: Ivana Ćirković and Deana Medic; Turkey: Serap Süzük Yildiz and Hüsniye Şimşek.

^a^ This designation is without prejudice to positions on status, and is in line with United Nations Security Council Resolution 1244/99 and the International Court of Justice Opinion on the Kosovo declaration of independence.
